# Simulation data for similarity of spray combustion processes in marine low-speed diesel engines

**DOI:** 10.1016/j.dib.2019.104837

**Published:** 2019-11-18

**Authors:** Xinyi Zhou, Tie Li, Yijie Wei

**Affiliations:** a State Key Laboratory of Ocean Engineering, Shanghai Jiao Tong University, PR China; b Collaborative Innovation Center for Advanced Ship and Deep-Sea Exploration, Shanghai Jiao Tong University, PR China

**Keywords:** Marine diesel engine, Similarity theory, Spray characteristics, Heat transfer loss, Emissions, Injection timing

## Abstract

Scaled model experiments are very useful for reducing time, cost and energy consumption in marine diesel engine development. This data article is based on the research work which examines the potential of scaled model experiments for marine low-speed diesel engines. Two engines of 340 and 520 mm bore diameters are employed to conduct this numerical scaling work based on three diesel combustion scaling laws. Data on similarity of peak swirl ratio, heat transfer losses, liquid and vapor penetration length, ignition delay, in-cylinder peak temperature, peak carbon monoxide (CO), peak hydrocarbon (HC) and carbon dioxide (CO_2_) emissions for various fuel injection timing are provided. The data in this paper are valuable reference for researchers or engineers who attempt to conduct scaled model experiments in marine diesel engine development.

**Table:** Specifications Table

Subject	Engineering
Specific subject area	Spray combustion processes in marine diesel engines
Type of data	Tables, figures
How data were acquired	Numerical simulation
Data format	Raw, simulated, analysed
Parameters for data collection	The similarity of swirl ratio, spray mixture formation, heat transfer, combustion characteristics and pollutant emissions are numerically studied using two engines of 340 and 520 mm bore diameters for three scaling laws at various fuel injection timings.
Description of data collection	The computational fluid dynamics (CFD) simulation model is established and calibrated against the experimental data from a marine low-speed diesel engine with 340 mm bore diameter [1]. Then, the potential of scaled model experiments for marine low-speed diesel engines is numerically studied with the baseline 340 mm-bore engine and an up-scaled 520 mm-bore engine at various injection timings. The data of accumulated heat transfer losses and pollutant emissions is normalized by fuel injection quantity for direct comparison between the large and small engines. The data of the liquid and vapor penetration length of the small engine is divided by the similarity ratio (*r*) for direct comparison with the large engine.
Data source location	State Key Laboratory of Ocean Engineering, Shanghai Jiao Tong University
Data accessibility	Data is with this article
Related research article	X.Y. Zhou, T. Li, Y.J. Wei, S.C. Wu, Scaling spray combustion processes in marine low-speed diesel engines, Fuel. 258 (2019). https://doi.org/10.1016/j.fuel.2019.116133.

**Table:** 

**Value of the Data** • This data explores the potential of scaled model experiments for marine low-speed diesel engines. • This data is valuable reference for researchers or engineers who attempt to conduct scaled model experiments in marine diesel engine development. • This data can be used to evaluate or correct the errors in the real applications of the scaled model experiments. • The data gives guidance on the selection of scaling laws in the real applications of the scaled model experiments.

## 1 Data

**Fig. 1:**
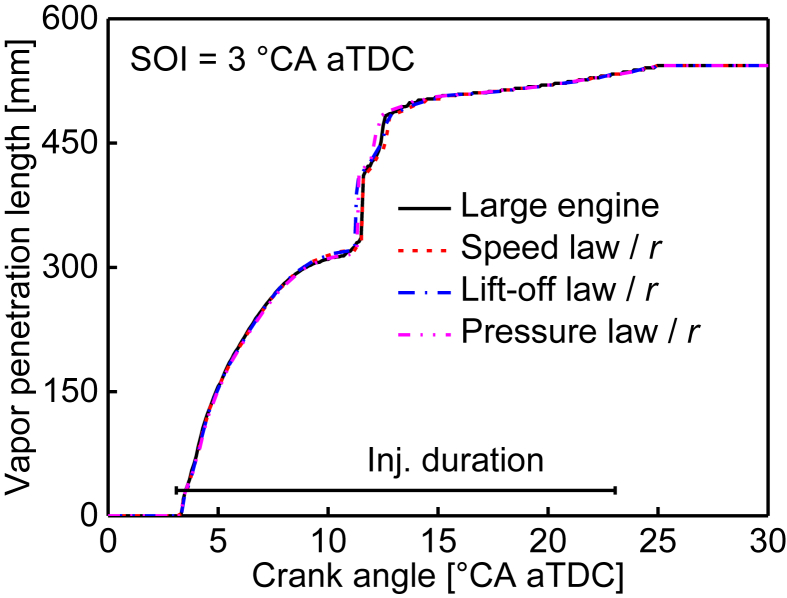
Similarity of the vapor penetration length evolution for the 3° CA aTDC injection timing. The vapor penetration length of the small engine is divided by the similarity ratio (*r*) for direct comparison with the large engine.

**Fig. 2:**
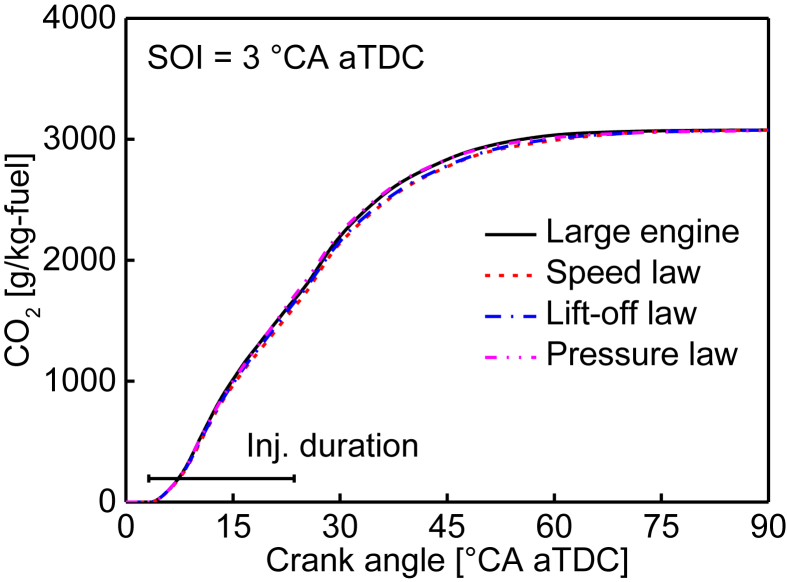
Similarity of the CO_2_ emissions for the 3° CA aTDC injection timing. The CO_2_ emissions are normalized by fuel injection quantity for direct comparison between the large and small engines.

Table 1 shows the similarity of the peak swirl ratio for various fuel injection timings. Table 2 represents the similarity of the accumulated heat transfer losses at the exhaust valve opening (EVO) timing for various fuel injection timings. Table 3 provides the similarity of the averaged liquid penetration length for various fuel injection timings, the averaged liquid penetration length is defined as the averaged value from 3 to 20 °CA after the start of injection. Fig. 1 shows the similarity of the vapor penetration length evolution for the 3° CA after top dead center (aTDC) injection timing. Table 4 and Table 5 describe the similarity of the ignition delay in crank angle and millisecond, respectively, the ignition delay is defined as the period from the start of injection to the 5% heat released timing. The similarity of in-cylinder peak temperature under various fuel injection timings is given in Table 6. Table 7 and Table 8 provide the similarity of the peak CO and HC emissions for various fuel injection timings, respectively. Fig. 2 shows the similarity of the CO_2_ emissions for the 3° CA aTDC injection timing.

**Table 1:** Similarity of the peak swirl ratio for various fuel injection timings.

Fuel injection timing (°CA aTDC)	Large engine (520 mm bore)	Small engine (340 mm bore)		
		Speed law	Lift-off law	Pressure law
−6	8.23	7.96	8.05	8.21
−3	8.07	7.83	7.94	8.03
0	7.90	7.64	7.78	7.92
3	7.77	7.47	7.60	7.54
6	7.57	7.28	7.33	7.44

**Table 2:** Similarity of the accumulated heat transfer losses in terms of J/mg-fuel at the EVO timing for various fuel injection timings. The accumulated heat transfer losses are normalized by fuel injection quantity for direct comparison between the large and small engines.

Fuel injection timing (°CA aTDC)	Large engine (520 mm bore)	Small engine (340 mm bore)		
		Speed law	Lift-off law	Pressure law
−6	3.94	4.24	4.26	4.26
−3	3.73	4.03	4.05	4.04
0	3.43	3.71	3.74	3.77
3	3.26	3.52	3.52	3.58
6	3.13	3.37	3.37	3.34

**Table 3:** Similarity of the averaged liquid penetration length in terms of mm for various fuel injection timings. The averaged liquid penetration length of the small engine is divided by the similarity ratio (*r*) for direct comparison with the large engine.

Fuel injection timing (°CA aTDC)	Large engine (520 mm bore)	Small engine (340 mm bore)		
		Speed law	Lift-off law	Pressure law
−6	87.46	88.90	88.43	87.96
−3	90.65	92.12	91.71	90.96
0	95.19	96.84	96.35	95.68
3	100.96	102.49	101.98	101.48
6	107.52	109.36	109.09	108.18

**Table 4:** Similarity of the ignition delay in crank angle in terms of °CA for various fuel injection timings.

Fuel injection timing (°CA aTDC)	Large engine (520 mm bore)	Small engine (340 mm bore)		
		Speed law	Lift-off law	Pressure law
−6	3.18	3.26	3.24	3.20
−3	3.15	3.24	3.22	3.18
0	3.17	3.25	3.23	3.19
3	3.22	3.30	3.28	3.24
6	3.29	3.37	3.36	3.32

**Table 5:** Similarity of the ignition delay in millisecond in terms of ms for various fuel injection timings.

Fuel injection timing (°CA aTDC)	Large engine (520 mm bore)	Small engine (340 mm bore)		
		Speed law	Lift-off law	Pressure law
−6	3.37	3.46	2.98	2.22
−3	3.35	3.43	2.96	2.21
0	3.37	3.45	2.97	2.21
3	3.41	3.50	3.02	2.25
6	3.49	3.58	3.09	2.31

**Table 6:** Similarity of the in-cylinder peak temperature in terms of K for various fuel injection timings.

Fuel injection timing (°CA aTDC)	Large engine (520 mm bore)	Small engine (340 mm bore)		
		Speed law	Lift-off law	Pressure law
−6	1747.87	1720.65	1729.98	1756.14
−3	1699.06	1663.46	1670.35	1690.68
0	1639.90	1621.76	1625.22	1644.30
3	1583.56	1562.19	1565.90	1585.19
6	1569.63	1545.30	1555.30	1569.96

**Table 7:** Similarity of the peak CO emissions in terms of g/kg-fuel for various fuel injection timings. The peak CO emissions are normalized by fuel injection quantity for direct comparison between the large and small engines.

Fuel injection timing (°CA aTDC)	Large engine (520 mm bore)	Small engine (340 mm bore)		
		Speed law	Lift-off law	Pressure law
−6	609.79	641.71	623.86	587.88
−3	640.90	677.99	650.26	619.56
0	685.75	715.90	699.06	658.81
3	724.29	759.03	733.30	696.72
6	762.90	787.69	771.72	747.79

**Table 8:** Similarity of the peak HC emissions in terms of g/kg-fuel for various fuel injection timings. The peak HC emissions are normalized by fuel injection quantity for direct comparison between the large and small engines.

Fuel injection timing (°CA aTDC)	Large engine (520 mm bore)	Small engine (340 mm bore)		
		Speed law	Lift-off law	Pressure law
−6	95.53	98.70	100.69	92.41
−3	102.43	105.65	106.45	104.86
0	106.34	112.69	109.70	105.54
3	103.11	105.11	105.34	104.09
6	110.63	114.76	114.52	114.82

### 1.1 Experimental design, materials, and methods

The CFD simulation model used in this paper is established and calibrated against the experimental data from a marine low-speed diesel engine with 340 mm bore diameter [1]. Then, the similarity of spray combustion processes in marine low-speed diesel engines is numerically studied with the baseline 340 mm-bore engine and an up-scaled 520 mm-bore engine at various injection timings. The geometry of the 520 mm engine is perfectly scaled with the well calibrated 340 mm diesel engine [1]. According to the three diesel combustion scaling laws described in Refs. [2,3], the operation conditions are described previously [1]. The engine speed is set constant between the large and small engines for the speed law, while the fuel injection pressure is set constant between the large and small engines for the pressure law. The same normalized fuel injection rate evolution is set between the 340 and 520 mm engine. In the present paper, the in-cylinder working processes between −70°CA aTDC to the EVO timing are simulated.
